# Evaluation of Dose Reduction Factors and Impact on Progression-Free Survival in Patients Treated with CDK 4/6 Inhibitors

**DOI:** 10.3390/jcm14041071

**Published:** 2025-02-07

**Authors:** Ali Kaan Güren, Murad Guliyev, Özkan Alan, Kıvanç Çadırci, İpek Naz Belevi, İlkay Gültürk, Emre Özge, Erkam Kocaaslan, Yeşim Ağyol, Pınar Erel, Burak Paçacı, Mustafa Alperen Tunç, Nargiz Majidova, Nadiye Sever, Abdussamet Çelebi, Rukiye Arıkan Erdoğan, Selver Işık, Nebi Serkan Demirci, Murat Sarı, Osman Köstek, İbrahim Vedat Bayoğlu

**Affiliations:** 1 Division of Medical Oncology, Department of Internal Medicine, Marmara University School of Medicine, Istanbul 34854, Turkey; erkamkocaaslan@gmail.com (E.K.); yesimagyol@gmail.com (Y.A.); pnarerell@gmail.com (P.E.); drpacaci@gmail.com (B.P.); m.alperentunc@gmail.com (M.A.T.); nergizmecidova91@gmail.com (N.M.); dr.nadya@hotmail.com (N.S.); abdussametcelebi@gmail.com (A.Ç.); dr_rukiyearikan@hotmail.com (R.A.E.); dr-selver83@hotmail.com (S.I.); drmuratsari@gmail.com (M.S.); osmankostek@yahoo.com (O.K.); drvebay@gmail.com (İ.V.B.); 2 Division of Medical Oncology, Department of Internal Medicine, Cerrahpasa Medical Faculty, Istanbul University-Cerrahpasa, Istanbul 34093, Turkey; drguliyev892@gmail.com (M.G.); ozkan.alan@hotmail.com (Ö.A.); nebiserkandemirci@hotmail.com (N.S.D.); 3 Department of Internal Medicine, Marmara University School of Medicine, Istanbul 34854, Turkey; kivanccadirci@gmail.com (K.Ç.); ipeknazbelevi97@gmail.com (İ.N.B.); 4 Department of Medical Oncology, Istanbul Research and Training Hospital, Istanbul 34093, Turkey; ilkayhulturk@gmail.com (İ.G.); emreozge87@gmail.com (E.Ö.)

**Keywords:** CDK 4/6 inhibitors, ribociclib, palbociclib, dose reduction

## Abstract

**Introduction:** CDK 4/6 inhibitors are effectively utilized among patients with hormone-positive, HER-2-negative metastatic breast cancer. The dose reduction for these patients varies between 35% and 57% across multiple trials. We aim to clarify the characteristics of patients who had dosage reduction and the effect of this reduction on survival outcomes. **Materials and Methods:** The study was designed as a retrospective, multicenter study. Patients who received CDK 4/6 inhibitors in the first-line and subsequent treatment settings were grouped based on dose reductions. Progression-free survival was compared between these groups, and factors influencing dose reduction were analyzed. **Results:** Multivariate logistic regression study demonstrated that patients aged 65 and older, with a Charlson CI score of 2 or higher, having metastases in three or more sites, and classed as normal weight, had greater dosage reductions. Dose reduction had no impact on progression-free survival (PFS) (*p* = 0.114 for first-line treatment, *p* = 0.528 for second and subsequent-line treatment; *p* > 0.05). **Conclusions:** Regarding the absence of disparity in progression-free survival between patients with dose reduction and those without, dose reduction should not be avoided in certain patient groups to ensure therapy continuity and mitigate potential adverse effects.

## 1. Introduction

Breast cancer is the most common cancer among women worldwide and is the second leading cause of cancer-related mortality in women [1]. Hormone receptor-positive, human epidermal growth factor receptor-2 (HER)-negative breast cancer constitutes almost 70% of all breast cancer cases [2]. In hormone-positive HER-2-negative metastatic breast cancer patients, ribociclib, palbociclib and abemaciclib have become standard first-line therapies following the PALOMA-2, MONALEESA-2, MONALEESA-7 and MONARCH-3 trials [3,4,5,6].

CDK 4/6 inhibitors induce numerous adverse effects, including neutropenia, tiredness, nausea, vomiting, rash, alopecia, pruritus, anemia, thrombocytopenia, hepatitis, extended QT interval and diarrhea. Patients administered ribociclib and palbociclib exhibit neutropenia rates of 80–95%, with 50–65% experiencing grade 3–4 neutropenia [7,8]. Among patients administered Abemaciclib, grade 3 neutropenia occurred in 18% and grade 4 neutropenia in 0.8%. In patients receiving abemaciclib in addition, 43% of these patients had grade 3 adverse effects, while 2.5% experienced grade 4 adverse effects [9]. Approximately 56–73% of patients on ribociclib and palbociclib therapy defer treatment at least once due to adverse effects, mainly neutropenia [10]. Dose decrease in individuals administered ribociclib and palbociclib ranges from 35% to 57% across various studies [11]. In patients administered abemaciclib, the predominant cause for dosage reduction was diarrhea; in the MONARCH-1 study, dose reduction occurred in 49% of patients, with 20% experiencing diarrhea [9].

The adverse effect profile of this medication, characterized by repeated postponements and dose reductions, has been thoroughly demonstrated by studies. Nonetheless, there is a paucity of studies examining which patients encounter worse pharmacological side effects and which patients necessitate more substantial dose reductions. From this perspective, we intend to elucidate the features of individuals undergoing dose reduction and to investigate the impact of dose reduction on survival within our patient population.

## 2. Materials and Methods

### 2.1. Study Population

Patients who received treatment in 3 different Medical Oncology Clinics between 1 January 2018 and 30 June 2024, diagnosed with hormone-positive HER-2-negative invasive breast cancer through histological examination, were included in the study. Patients underwent treatment with Ribociclib or Palbociclib during the metastatic stage.

Ribociclib and palbociclib were administered at doses of 600 mg and 125 mg daily, days 1–21 of each 28-day cycle, respectively. Aromatase inhibitors (anastrozole 1 mg or letrozole 2.5 mg daily) or fulvestrant 500 mg on days 1, 15, 29, and once monthly were administered as combined endocrine therapy. Dose reduction was defined as a reduction in ribociclib dose from 600 mg to 400 mg or 200 mg and palbociclib dose from 125 mg to 100 mg or 75 mg.

### 2.2. Study Design and Data Collection

The study was designed as a retrospective, multicenter study. The data of the patients were analyzed using patient records and the hospital electronic information system. Patients were divided into two groups, as those who underwent dose reduction at least once due to side effects (dose-reduced group) and those who did not (non-dose-reduced group). Age, menopausal status, body mass index (BMI), body surface area (BSA), Charlson comorbidity index (CCI), progesterone and HER-2 receptor status, metastatic sites and number of metastatic sites were compared between these two groups. Subsequently, patients administered CDK 4/6 inhibitors in the initial series and in subsequent series were distinctly categorized into groups with and without dose reduction, and progression-free survival (PFS) was compared between these groups. Performance scores were calculated using the Eastern Cooperative Oncology Group Performance Score (ECOG PS). Comorbidity score was calculated according to the original CCI, the 19-item version. BMI was calculated using the formula kilogram/meter^2^ and BSA was calculated using the formula 0.20247 × Height(m)0.725 × Weight(kg)0.425. Adverse events were performed according to the Common Terminology Criteria for Adverse Events (CTCAE) version 5.0.

### 2.3. Statistical Analysis

SPSS version 22.0 (IBM corp., Armonk, NY, USA) was used for all statistics. PFS was calculated as the time in months from the patient’s first dose of treatment to disease progression or to the day of the last visit if the patient was still receiving treatment. If the patient died while on treatment, the last date was considered as the date of death. When evaluating the study data, the conformity of the parameters to normal distribution was evaluated by Shapiro–Wilk test. Categorical variables were calculated using chi-square, and 95% confidence intervals (CIs) were calculated using the Brookmeyer and Crowley method. Kaplan–Meier survival analysis was used for survival analyses and survival differences between groups were compared by log-rank test. Logistic regression analysis (forward-conditional) was used in the multivariate evaluation of variables affecting drug dose reduction. Significance was evaluated at *p* < 0.05 level.

## 3. Results

The study included 474 female patients diagnosed with hormone-positive HER-2-negative breast cancer. The baseline characteristics of the patients are summarized in Table 1. The median age of the patients was 56 years, ranging from 27 to 87 years, and 148 patients were pre-menopausal. 312 patients had a BMI of 25 and higher and 328 patients had a BSA of 1.7 and higher. The most common subtype was invasive ductal carcinoma, with 76.9%. All patients were estrogen receptor-positive and 87 (%18.4) patients were progesterone receptor-negative. A total of 30.8% of patients presented with metastases at three or more sites.

A total of 343 patients received ribociclib, while 131 patients were treated with palbociclib. Aromatase inhibitors were administered to 314 patients, while fulvestrant was given to 160 patients. In the first series, 307 patients received CDK 4/6 inhibitors, whereas 167 patients were treated with them in the second and subsequent series. Neutropenia of all grades was observed in 90.9% of patients, with 40.1% presenting with grade 3 and 8.6% with grade 4 neutropenia. Nine patients (1.8%) exhibited thrombocytopenia, while seven patients (1.4%) presented with anemia. In the eighth month of treatment, one patient developed myelodysplastic syndrome (MDS) accompanied by persistent pancytopenia. A Grade 3–4 extended QT interval was noted in 12 individuals, all of whom were administered ribociclib. Treatment characteristics and adverse events are outlined in Table 2.

**Figure 1:**
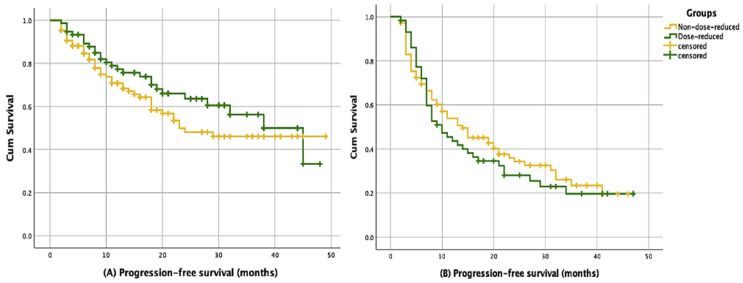
(**A**): PFS of patients receiving CDK 4/6 inhibitors as first-line treatment, (**B**): PFS of patients receiving CDK 4/6 inhibitors in the second- and subsequent-line treatments.

The first dose reduction was implemented at a mean of 4.44 ± 0.52 months, while the second dose reduction occurred at a mean of 7.33 ± 1.17 months. The median follow-up time for the entire cohort was 23.1 months (IQR 13.2–33.4). The median PFS for first-line therapy was 29.4 months (95% CI, 19.1–38.8). In this group, the median PFS was 24.8 months (95% CI, 17.1–29.9) for patients without a dose reduction and 37.1 months (95% CI, 25.4–50.5) for those with a dose reduction. No statistically significant difference was found between the two groups (*p* = 0.11). The median PFS for patients undergoing therapy in the second and subsequent lines was 13.1 months (95% CI, 9.6–16.3). In this patient group, the median PFS was 14.4 months (95% CI, 7.8–20.1) for patients without a dose reduction and 10.6 months (95% CI, 5.9–14.2) for those with a dose reduction. There was no statistically significant difference between two groups (*p* = 0.52). Survival results are presented in Table 3. Figure 1 displays the Kaplan–Meier curves for all groups.

The Hosmer and Lemeshow test was used for compatibility with logistic regression analysis and *p* = 0.753 (*p* > 0.05). Multivariate logistic regression analysis was performed upon evaluation as compatible for the analysis. Patients over 65 years of age had more dose reductions than patients under 65 years of age (OR 1.906, 95%CI 1.132–3.209, *p* = 0.015). In the group with BMI higher than normal, fewer reductions were performed compared to normal (OR 0.575 %95CI 0.332–0.995 *p* = 0.048). In the group with a CCI score of 2 and higher, more dose reductions were performed compared to the group with 0 or 1 (OR 1.944 %CI 1.128–3.350, *p* = 0.017). In the group with three or more metastasis sites, dose reduction was more frequent than in the group with fewer metastasis sites (OR 2.325 %CI 1.380–3.917, *p* = 0.002). There was no significant relationship between dose reduction and treatment lines, menopausal status, BSA, de novo metastasis, progesterone receptor status, HER-2 receptor status and metastasis sites. These results are summarized in Table 4.

## 4. Discussion

Our analysis revealed that dose reduction was more prevalent among patients aged 65 and older, with a Charlson Comorbidity Index score of 2 or higher, presenting metastases in three or more sites, and classified as normal weight. Furthermore, when patients administered CDK 4/6 inhibitors in the initial series were analyzed separately from those in the second and subsequent series, no statistically significant difference in PFS was observed between those who experienced dose reduction and those who did not (*p* = 0.114 for first-line treatment, *p* = 0.528 for second- and subsequent-line treatment; *p* > 0.05).

In the MONALESSA 2-3-7 investigations, dose reductions were implemented at rates of 54%, 38%, and 37%, respectively, due to adverse effects. In the pooled analysis encompassing the MONALESSA 2-3-7 investigations conducted by Burris et al., the PFS of patients who experienced dose decrease was comparable to that of those who did not. In the aggregated post hoc analysis of the PALOMA 1-2-3 investigations, dosage reduction occurred in 36.9% of all patients, and no difference in efficacy for palbociclib was observed between those with and without dose reduction [12]. Our study, employing real-world data, demonstrated no statistically significant difference in PFS between patients treated with ribociclib and palbociclib throughout first line, second line, and subsequent lines, regardless of whether they received dosage reduction. Additionally, logistic regression analysis indicated no statistically significant difference in dosage reduction between patients using CDK4/6 treatment in the first or second line (35.8% vs. 35.3%, *p* = 0.877). In the MONARCH-2 study, dose reduction was observed in 42.9% of patients, but in the MONARCH-3 study, it was 43.4%, mostly attributable to adverse effects, predominantly diarrhea [6,13,14]. Rugo et al. observed no disparity in PFS between people who underwent dosage reduction and those who did not.

Previous studies indicated that groups with a BMI exceeding the usual range exhibited reduced instances of neutropenia and lower dosage reductions compared to the normal group [15,16]. Consistent with the literature, the group with a BMI of 25 or below had a greater incidence of dose decrease in our trial. The small group of underweight patients in our study hindered our ability to compare the dosage reduction between normal weight and underweight individuals. We are of the opinion that dose reduction should not be avoided in the event of potential adverse effects, particularly in patients who are BMI of 25 or below.

With advancing age, our bone marrow reserve declines [17,18]. Clinical trials and real-world data investigations of ribociclib and palbociclib have revealed a heightened incidence of dose reductions among patients aged over 65 years [19,20,21]. Research data and many studies indicate that heightened vigilance is necessary about side effects, especially in geriatric people. Regarding the absence of survival differential, dose reduction should be considered, particularly for elderly patients suffering from adverse effects.

In post hoc analyses of the PALOMA-2 study, the rates of dose reduction were similar between patients with and without preexisting conditions. The rate of dose reduction was found to be higher only in patients with four or more preexisting conditions [22]. In our study, it was observed that dose reduction was performed more in patients with a Charlson CI score of 2 and above. Given the bone marrow suppression resulting from chronic diseases, drug–drug interactions, and drug-induced bone marrow toxicities, we propose that adverse effects associated with CDK 4/6 inhibitors may have manifested more frequently, even in the absence of direct drug–drug interactions.

The limitations of our investigation encompass its retrospective nature and the inability to precisely characterize grade 1–2 side effects that are not demonstrated in laboratory results. The restricted patient population hindered a more comprehensive examination of age (>75), BMI (<18), and BSA. Moreover, in previous series, we could not discern differences between patients who underwent chemotherapy or treatments potentially inducing bone marrow suppression and those who did not. Our study provides significant insights for physicians, featuring a multicenter design, a substantial patient group, and real-world data. Our study identifies patient categories that should exercise greater caution regarding the encountered adverse effects.

In conclusion, patients aged 65 and older, with a Charlson CI score of 2 or higher, having metastases in three or more sites, and classed as normal weight, had greater dosage reductions. No difference in progression-free survival was observed between patients experiencing side effects from ribociclib and palbociclib who received dose reduction and those who did not. Especially in these patient groups, more attention should be paid to treatment side effects. In the future, studies examining the treatment outcomes of patients started with a lower initial dose are needed to avoid possible side effects and dose deferrals.

## Figures and Tables

**Table 1:** Baseline clinic and pathologic characteristics of patients.

	Total Patiens (*n* = 474)
Age (min–max)	56 (27–87)
Menopausal status (%)	
Pre-menopausal	148 (31.2)
Post-menopasual	326 (68.8)
BMI (%)	
<25	142 (31.1)
≥25	312 (68.9)
Unknown	20
BSA (%)	
<1.7	126 (27.8)
≥1.7	328 (72.2)
Unknown	20
ECOG PS (%)	
PS 0–1	452 (95.4)
PS ≥ 2	22 (4.6)
Charlson CI (%)	
0–1	369 (77.8)
≥2	105 (22.2)
De novo metastatic (%)	
Yes	271 (57.2)
No	203 (42.8)
Histological Subtype (%)	
Invasive ductal carcinoma	334 (76.9)
Invasive lobular carcinoma	47 (10.8)
Others	53 (12.2)
Unknown	40
Progesterone receptor status (%)	
Positive (≥%1)	387 (81.6)
Negative (<%1)	87 (18.4)
HER-2 receptor status (%)	
Positive (1 or 2 + and fısh negative)	91 (19.2)
Negative	383 (80.8)
Number of metastatic sites (%)	
<3	299 (69.2)
≥3	133 (30.8)
Bone metastases only (%)	
Yes	139 (32.1)
No	293 (67.9)
Liver or lung metastases (%)	
Yes	199 (46.1)
No	233 (53.9)
Brain metastases (%)	
Yes	409 (94.6)
No	23 (5.4)

BMI: body mass index, BSA: body surface area, Charlson CI: Charlson comorbidity index, ECOG PS: Eastern Cooperative Oncology Group Performance Score, HER-2: human epidermal growth factor receptor-2.

**Table 2:** Treatment characteristics and adverse events.

	Total Patiens (*n* = 474)
CDK 4/6 inhibitors (%)	
Ribociclib	343 (72.4)
Palbociclib	131 (27.6)
Combination endocrine therapy (%)	
Aromatase inhibitors	314 (66.2)
Fulvestrant	160 (33.8)
Treatment Line (%)	
First line	307 (64.8)
Second and subsequent lines	167 (35.2)
Dose reduction (%) (*any cause*)	
No	305 (64.3)
Yes	169 (35.7)
One time	140 (29.5)
Two times	29 (6.1)
Neutropenia (%) (any grade)	431 (90.9)
Neutropenia (%) (grade 3–4)	231 (48.7)
Prolonged QT interval (%) (grade 3–4)	12 (2.5)
Thrombocytopenia (%) (grade 3–4)	9 (1.8)
Anemia (%) (grade 3–4)	7 (1.4)
Hepatobiliary toxicity (%) (grade 3–4)	6 (1.2)
Diarrhea (%) (grade 3–4)	3 (0.6)
Stomatitis (%) (grade 3–4)	1 (0.2)
Pulmonary embolism (%) (grade 3–4)	1 (0.2)

**Table 3:** Median progression-free survival of patients treated in the first lines and in the second and subsequent lines.

	*n* (%)	Median PFS (%95 CI) Months	*p*
First-line treatment			
Non-dose-reduced	197 (64.1)	24.8 (17.1–29.9)	
Dose-reduced	110 (35.8)	37.1 (25.4–50.5)	0.114
Overall	307	29.4 (19.1–38.8)	
Second- and subsequent-line treatment			
Non-dose-reduced	108 (64.6)	14.4 (7.8–20.1)	
Dose-reduced	59 (35.3)	10.6 (5.9–14.2)	0.528
Overall	167	13.1 (9.6–16.3)	

CI: confidence interval, PFS: progression-free survival.

**Table 4:** Comparison of dose-reduced group and non-dose-reduced group by multivariate logistic regression analysis.

	B	OR	OR %95CI	*p*
Age (<65 vs. ≥65)	0.645	1.906	1.132–3.209	**0.015**
Menopausal status	0.070	1.072	0.627–1.833	0.798
BMI (<25 vs. ≥25)	−0.554	0.575	0.332–0.995	**0.048**
BSA (<1.7 vs. ≥1.7)	0.148	1.159	0.711–1.889	0.553
Charlson CI (0–1 vs. 2+)	0.665	1.944	1.128–3.350	**0.017**
De novo metastasis	−0.351	0.115	0.455–1.090	0.115
Treatment lines (1. vs. 2 and subsequent line)	−0.640	0.938	0.603–1.459	0.877
Progesterone receptor status (<%1 vs. ≥%1)	−0.092	0.912	0.521–1.596	0.746
HER-2 receptor status (0 vs. 1 or 2+)	0.247	1.281	0.753–2.178	0.361
Number of metastatic sites (<3 vs. **≥3)**	0.844	2.325	1.380–3.917	**0.002**
Bone metastases only	0.222	1.248	0.656–2.374	0.499
Liver or lung metastases	−0.343	0.710	0.406–1.242	0.230
Brain metastases	−1.006	0.366	0.125–1.070	0.066

BMI: body mass index, BSA: body surface area, Charlson CI: Charlson comorbidity index, HER-2: human epidermal growth factor receptor-2.

## Data Availability

The data that support the findings of this study are not publicly available due to privacy reasons but are available from the corresponding author.
